# Supplementary material for: The Genomic Architecture of Adaptation to Larval Malnutrition Points to a Trade-off with Adult Starvation Resistance in Drosophila
Source: Mol Biol Evol. 2021 Mar 2;38(7):2732–49. doi: 10.1093/molbev/msab061 (PMC8233504; doi:10.1093/molbev/msab061)
Supplement: msab061_Supplementary_Data [file msab061_supplementary_data.zip › Kawecki_etal_MalnutritionGenomics_SI_document.pdf]

The genomic architecture of adaptation to larval malnutrition points to a trade-off with adult starvation resistance in *Drosophila* – Supplementary Information

TADEUSZ J. KAWECKI, BERRA ERKOSAR, CINDY DUPUIS, BRIAN HOLLIS, R. CRAIG STILLWELL and MARTIN KAPUN

Population genetic analyses

Chromosome-wide patterns of within-population genetic variation were similar between the regimes. We found no differences between Selected and Control populations for genome-wide estimates of π and Watterson's θ on any autosomal arm (Mann Whitney U tests; all $W < 25$, $P > 0.05$). There was a marginally significant deviation on the *X* for π (Mann Whitney U tests; $W = 31$, $P = 0.041$), but not for θ. These results are complementary to the estimates of N_e and indicate overall similar effective population sizes in both regimes. Chromosome-wide π values ranged from 0.0004 to 0.0012 for the *X* and 0.0021 to 0.0025 for autosomes respectively (see Supplementary Table S1) which is markedly lower than previous estimates from other natural and experimental *D. melanogaster* populations (see Figure S1) based on pooled sequencing data. Our experimental design that allowed only 200 adults per replicate populations to contribute to next generation and the long course of the experiment in comparison to other experimental evolution studies may have resulted in loss of genetic variation due to drift. When investigating window-wise chromosomal patterns of genetic variation, we identified several genomic regions with statistically significant differences in π and θ with respect to regimes (see Figure S1). Curiously, we found chromosomal regions with strong reductions of genetic variation not only in Selected, but also in consistent across Control populations (see Figure S1).

Chromosomal inversions

We tested to which extent genetic diversity could be influenced by chromosomal inversions, which have been shown to have a strong impact on recombination rates and genetic variation (Kapun and Flatt 2018). Based on inversion-specific marker SNPs, the cosmopolitan inversions *In(2L)t, In(2R)NS, In(3L)P, In(3R)C, In(3R)Mo, In(3R)K* and *In(3R)Payne* were completely absent or segregated at very low frequencies (< 5%) in all populations. These findings may either indicate that inversion were at low frequencies or completely absent at the beginning of the selection experiment or alternatively decreased in frequency during the course of the experiment. Such a pattern would be consistent with similar findings from other lab-based experimental evolution studies, which all report that common cosmopolitan inversion rapidly decrease in frequency when maintained under laboratory conditions (Kapun et al. 2014). We therefore conclude that inversions only played a limited role during the evolutionary process in our experiment.

Supplementary Figures

Supplementary Figure S1

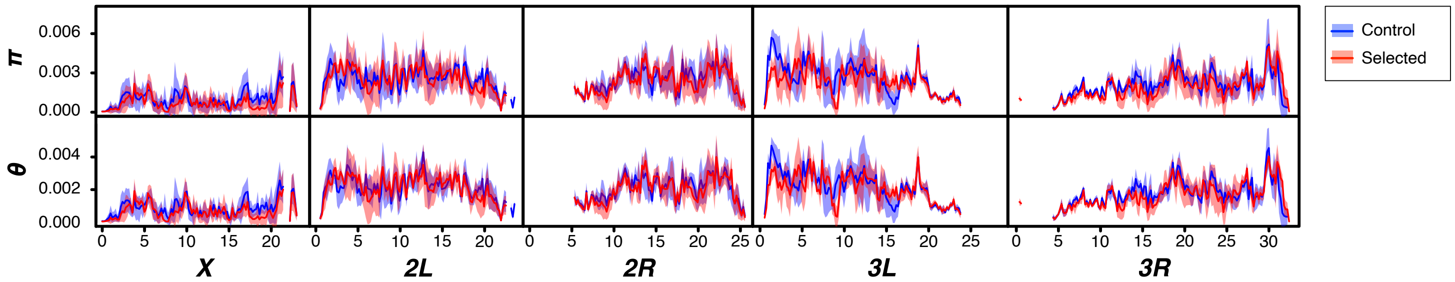

Supplementary Figure S1: Population genetic analysis of candidates for selection. The panels show chromosome-wise averages for the population genetic estimators π, θ and Tajima's D in non-overlapping windows of 200 kbp size. Similar to Figure 2, solid lines and semi-transparent polygons show means and standard deviations for these estimators, respectively, that were calculated from six selected (red) and six control (blue) replicate populations.

Supplementary Figure S2

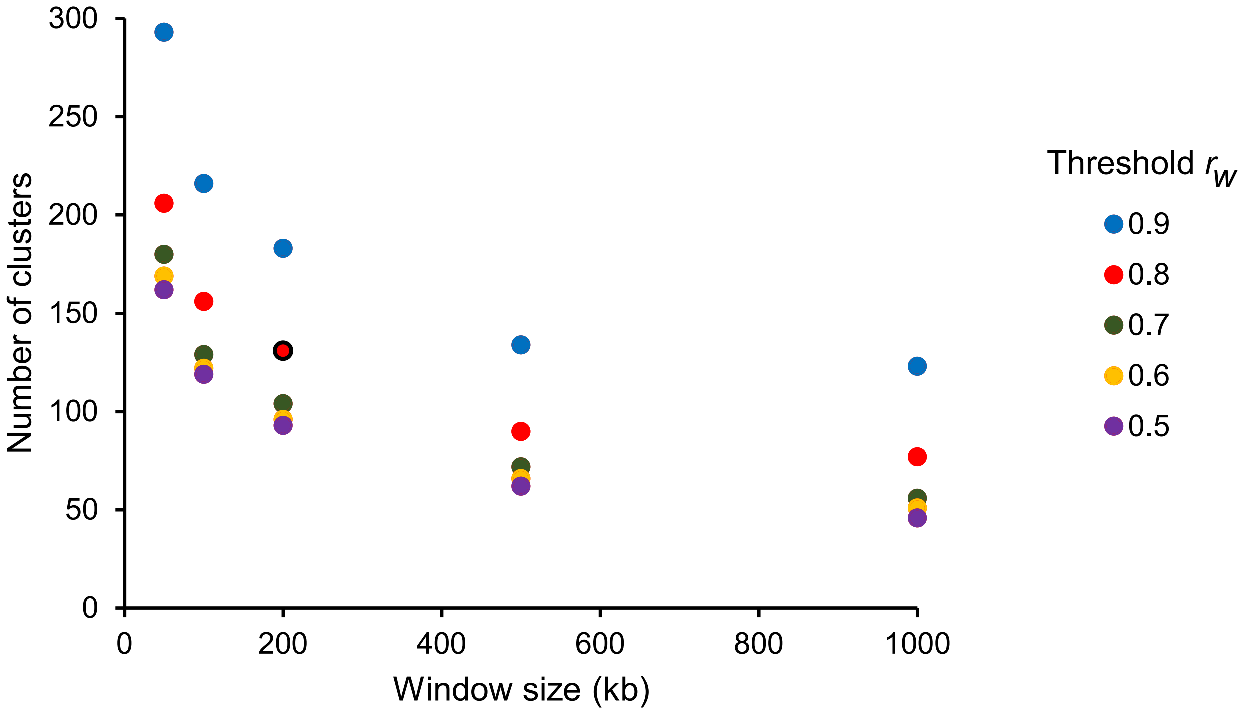

Supplementary Figure S2. The number of putatively independently evolving candidate SNP clusters as a function of parameters used to delineate them, the window size and threshold correlation. To delineate the clusters, we examined within-regime correlations in allele frequencies r_w between candidate SNPs within a "window" of a given size to the left of the midpoint between each pair of neighboring candidate SNPs and the candidate SNPs in the analogous window to the right of the midpoint. If none of these correlations was greater than the threshold value, the midpoint was recognized as a boundary between two adjacent clusters. If the distance between the neighboring candidate SNPs was greater than the window size, the SNPs were likewise attributed to different clusters. Otherwise, the two neighboring SNPs were attributed to the same cluster. This procedure was repeated for each pair of adjacent candidate SNPs.

A larger window or lower threshold correlation results in fewer clusters. One reason is that a larger window allows detection of longer-range LD, and a lower threshold correlation would be sensitive to less-than-perfect LD. However, the same changes in parameters also increase the likelihood of “false positives” in the sense of grouping in one cluster SNPs that in fact evolved independently. This is because some pairs of SNPs are likely to be highly correlated by chance - even for pairs of SNPs on different chromosomes 0.7 % show $r_w > 0.8$ and this increases to 3.7% for $r_w > 0.6$ (Fig. 3A). The window size of 200 kb and threshold correlation 0.8 (black encircled symbol) were chosen to delineate clusters reported in the main text because they resulted in the distribution of r_w between pairs of candidate SNPs in different clusters on the same chromosomal arm (Fig 3C) being very similar to the distribution of r_w for candidate SNPs on different chromosomes (Fig 3A).

Supplementary Figure S3

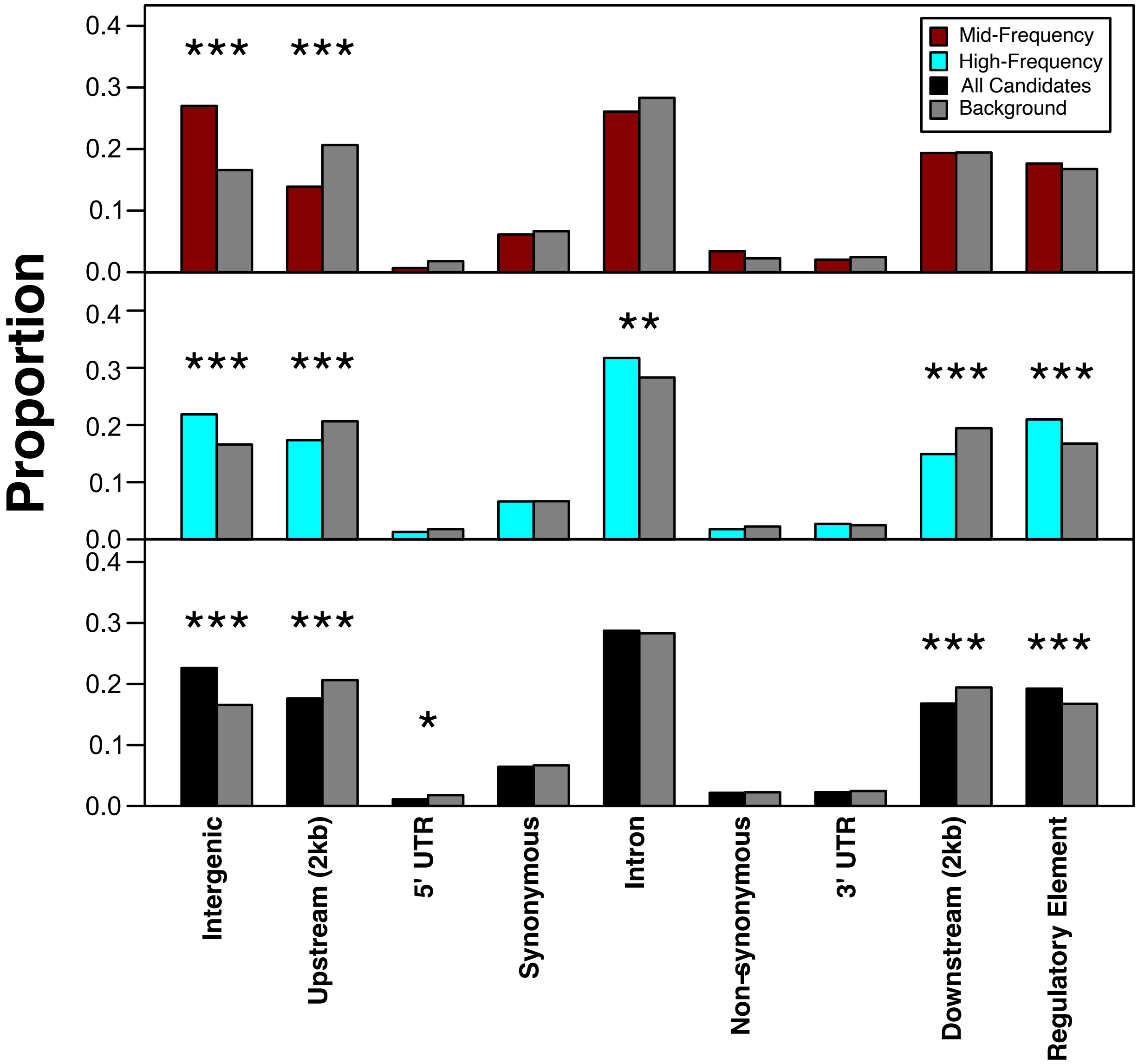

Supplementary Figure S3. Over- and underrepresentation of candidate SNPs in genomic functional classes. Histograms showing the proportion of candidates for balancing selection (in orange), directional selection (in cyan) and for all candidates (in black) in functional SNP effect classes, which were assessed by SNPeff annotation and based on the Redfly database. Expected proportions were estimated from all non-candidate SNPs and are highlighted in grey. Significant over- or underrepresentation of selected SNPs was assessed by chi-square tests with Bonferroni corrections (corrected α =0.006) comparing the counts of candidate and non-candidate SNPs in a given feature against the remaining candidate and non-candidate SNPs. ** $P < 0.01$; *** $P < 0.001$

Supplementary Figure S4

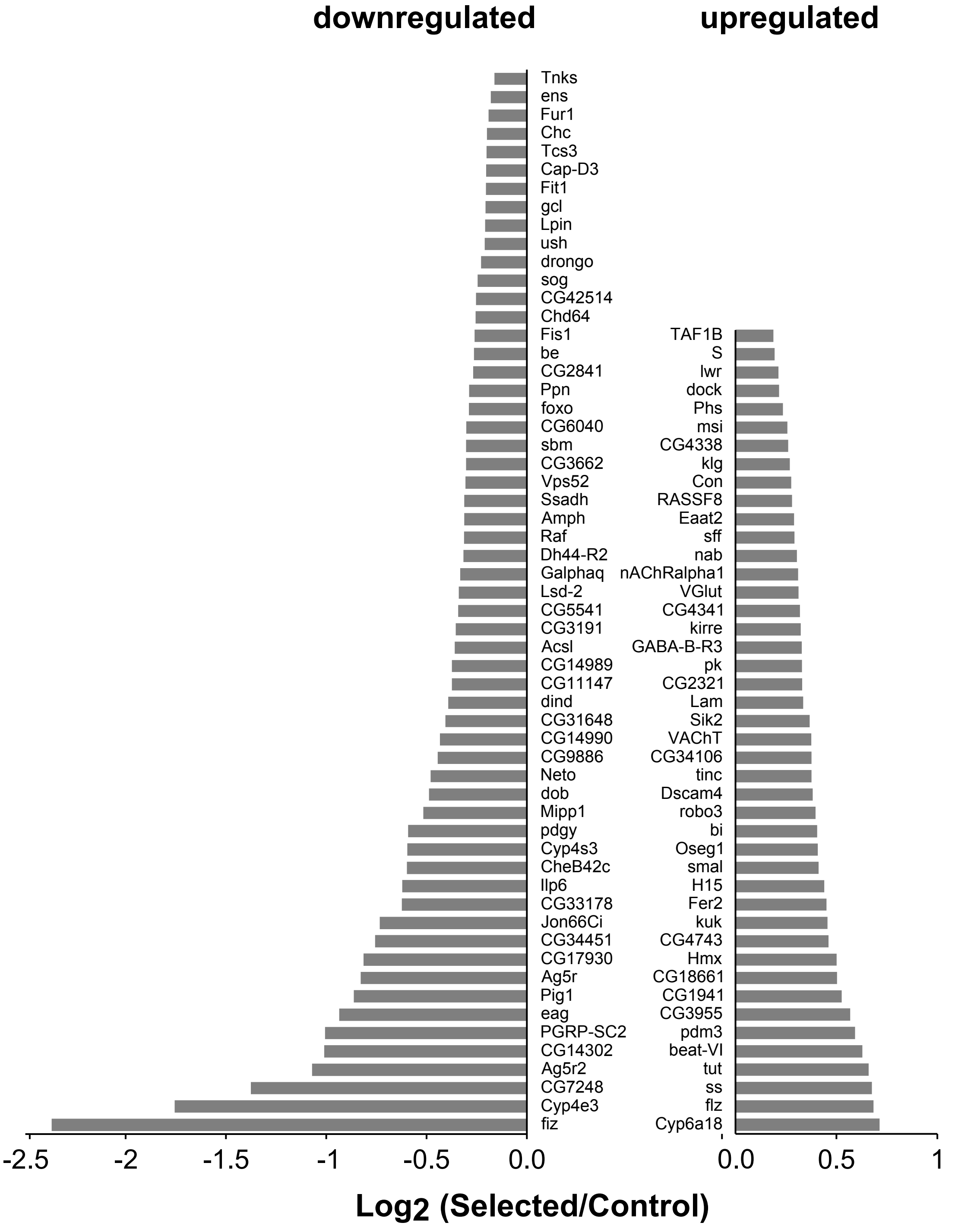

Supplementary Figure S4. Expression patterns of candidate genes shared across genomic and transcriptomic analyses. Bar plots showing the log_2-fold differential expression between Selected and Control populations for genes which have been identified as candidates both in the genomic analyses of this study and transcriptomic analyses in Erkosar et al. (2017). Left bars show downregulated and the right bars upregulated candidate genes.

Supplementary Figure S5

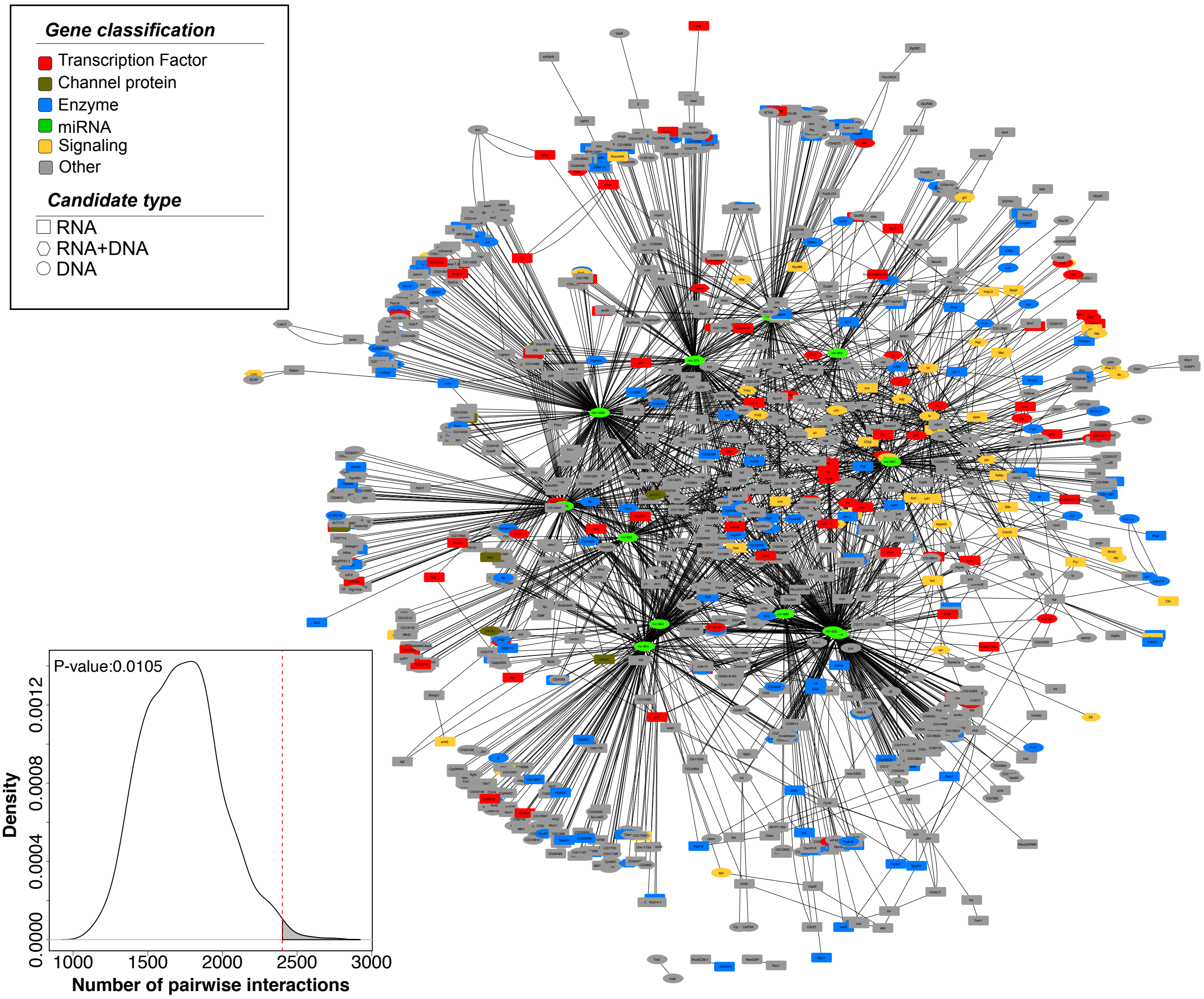

Supplementary Figure S4: Interaction among all candidate genes. A gene network showing interactions among all genomics candidates and genes which show significant differential expression among selection regimes (Erkosar et al. 2017). Colors depict a rough classification of genes according to gene function and symbol shape highlight the data source of each candidate (see also Supplementary Table S5 for a complete list of genes). The subplot at the bottom left depicts the distribution of numbers of pairwise interaction in 1000 randomly drawn non-candidate gene sets, that matched the true dataset in number of candidates, gene lengths and gene classifications. The vertical red line highlights the number of interactions in the "true" datasets and was used as a threshold to calculate empirical *P*-value shown in the top-right corner.
